# Exceptionally clean single-electron transistors from solutions of molecular graphene nanoribbons

**DOI:** 10.1038/s41563-022-01460-6

**Published:** 2023-02-02

**Authors:** Wenhui Niu, Simen Sopp, Alessandro Lodi, Alex Gee, Fanmiao Kong, Tian Pei, Pascal Gehring, Jonathan Nägele, Chit Siong Lau, Ji Ma, Junzhi Liu, Akimitsu Narita, Jan Mol, Marko Burghard, Klaus Müllen, Yiyong Mai, Xinliang Feng, Lapo Bogani

**Affiliations:** 1 Center for Advancing Electronics Dresden & Faculty of Chemistry and Food Chemistry, Technische Universität Dresden, Dresden, Germany; 2 School of Chemistry and Chemical Engineering, Shanghai Jiao Tong University, Shanghai, China; 3 Department of Materials, University of Oxford, Oxford, UK; 4 Max Planck Institut für Festkörperforschung, Stuttgart, Germany; 5 Max Planck Institut für Polymerforschung, Mainz, Germany; 6 Max Planck Institute of Microstructure Physics, Halle, Germany; 7 Present Address: Institute of Materials Research and Engineering, Singapore, Singapore; 8 Present Address: School of Physics and Astronomy, Queen Mary University of London, London, UK

**Keywords:** Electronic properties and devices, Synthesis of graphene

## Abstract

Only single-electron transistors with a certain level of cleanliness, where all states can be properly accessed, can be used for quantum experiments. To reveal their exceptional properties, carbon nanomaterials need to be stripped down to a single element: graphene has been exfoliated into a single sheet, and carbon nanotubes can reveal their vibrational, spin and quantum coherence properties only after being suspended across trenches^1–3^. Molecular graphene nanoribbons^4–6^ now provide carbon nanostructures with single-atom precision but suffer from poor solubility, similar to carbon nanotubes. Here we demonstrate the massive enhancement of the solubility of graphene nanoribbons by edge functionalization, to yield ultra-clean transport devices with sharp single-electron features. Strong electron–vibron coupling leads to a prominent Franck–Condon blockade, and the atomic definition of the edges allows identifying the associated transverse bending mode. These results demonstrate how molecular graphene can yield exceptionally clean electronic devices directly from solution. The sharpness of the electronic features opens a path to the exploitation of spin and vibrational properties in atomically precise graphene nanostructures.

## Abstract (web summary)

Molecular graphene nanoribbons hold promise for quantum experiments in single-electron transistors but require improvements in their debundling. Here, the authors demonstrate ultra-clean transport devices by enhancing nanoribbon solubility via bulky groups on the nanoribbon edges.

## Main

Molecular graphene nanoribbons (MGNRs)^7–12^ can be chemically synthesized with a degree of control over the edges and topology^7,13,14^ that is vastly superior to what can be achieved with top-down nanofabrications^2,4,8–10^. The molecular precision of the edges frees these systems from spurious scattering, possibly enabling intrinsic quantum phenomena to become manifest^8,9,11,12,15–17^, and spin coherence times up to microseconds have been observed^17^. Conditions are thus ripe to use a single MGNR to exploit spin and topological phenomena in quantum electron transport, but only single-electron transistors with a certain level of cleanliness, where all states can be properly accessed, can be used for quantum experiments. However, the integration of MGNRs into electronic nanodevices is still in its infancy, and devices with carpets of nanoribbons or surface-grown MGNRs are intrinsically ill-suited for the observation of quantum transport phenomena. Carbon nanotubes (CNTs) have experienced similar issues: surfactants that improve solubility are detrimental to the electronic and optical properties, and a step change was achieved only with the growth of ultra-clean CNT devices across trenches, where vibrational and spin effects could be identified and studied.

The geometry of MGNRs is, intrinsically, dominated by edges, and this offers a singularly simple way towards extreme cleanliness, without dedicated fabrication steps or surfactants: substitutions by groups that are regularly spaced along the MGNR edges, neutrally charged and covalently bonded, so that they improve solubility without impacting the electronic properties. Previous synthetic attempts have led to dispersible MGNRs^5,6^, but the degree of debundling necessary for electronic devices is still undemonstrated.

**Fig. 1:**
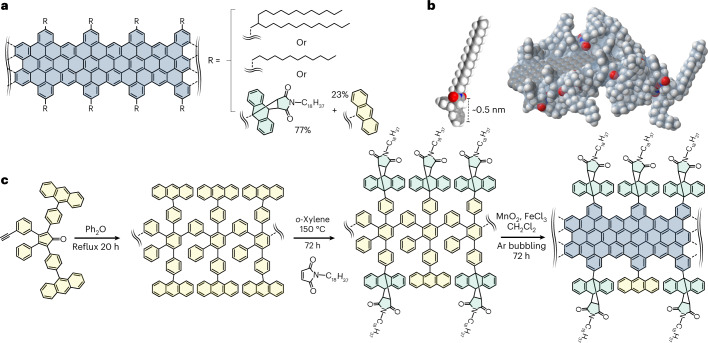
Synthetic design. **a**, Structure of a MGNR backbone, showing its atomically precise width and edge structure. The different side functionalizations are highlighted for two traditional MGNRs: MGNR **1**, with alkyl side chains for solubilization (top), and MGNR **2**, with bulky groups that hinder *π*-stacking (bottom). **b**, Space-filling model of a segment of **2** showing the Connolly solvent-excluded surface in light blue and the bulky edge groups used to suppress *π*-stacking. Red atoms are oxygen, grey are carbon, white are hydrogen and blue are nitrogen **c**, Synthetic route to obtain MGNR **2** (details in Methods and Supplementary Information).

Our strategy here is to demonstrate extreme electronic cleanliness by the debundling produced via chemical design of the MGNR side groups. To show different behaviours, we compare MGNRs with the same aromatic backbone but with different substitutions along the edges. Our backbone of choice has a width *w* = 1.13 nm (refs. ^2^^,3,14–16^), and a wide distribution of lengths *l* = 10–371 nm is found. MGNRs usually^3^ sport alkyl chains per repeating unit, such as the groups in **1a** and **1b**, used here for comparison (Fig. 1a). To produce soluble nanoribbons we introduce bulky three-dimensional side groups that have a diameter of ~0.5 nm, much larger than the typical distance between *π*-stacked graphitic layers, ~0.3 nm (Fig. 1b). The synthetic route sees first the creation, via Knoevenagel condensation, of a diene and a dienophile, and then removal of the triisopropylsilyl protecting groups to afford the monomer (**2a**; Supplementary Figs. 1–7). AB-type Diels–Alder polymerization of **2a** leads to a polyphenylene precursor pendent with anthracene functional groups (**2b**; Supplementary Fig. 8). Subsequently, Diels–Alder ‘click’ cycloaddition with *N*-octadecylmaleimide produces a polyphenylene (**2c**) with the desired bulky groups on the sides (Supplementary Fig. 9). Intramolecular oxidative cyclodehydrogenation eventually forms the aromatic backbone and yields **2** (Fig. 1c and Supplementary Figs. 10–12).

**Fig. 2:**
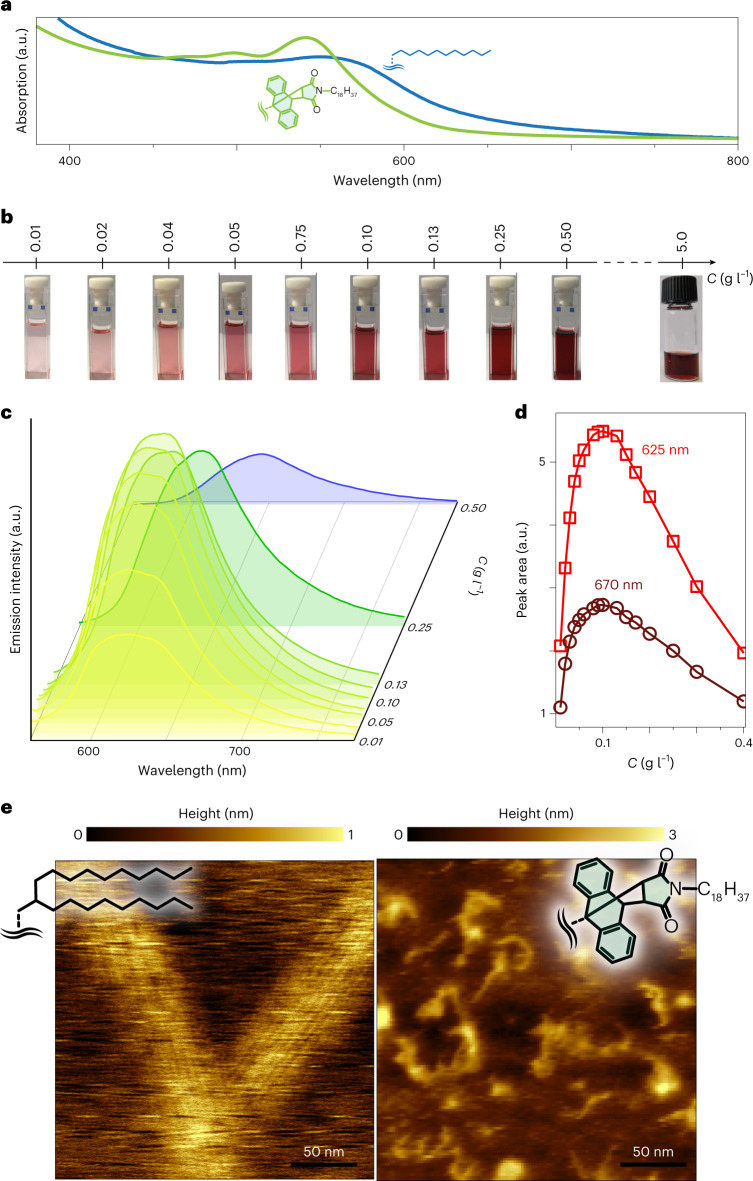
Debundling of the molecular nanoribbons. **a**, Normalized UV–visible absorption spectra of **1** (blue) and **2** (green) in chloroform. The insets show the molecular structure. **b**, Photographs of the chloroform solutions of MGNR2 at different concentrations, showing dispersibility. **c**, Evolution of the photoluminescence spectra of **2** in chloroform with excitation of 541 nm on increasing the concentration (*C*), with photographs showing the homogeneous solubilization of **2** in chloroform. **d**, Relationship between the photoluminescence peak areas and *C* for the two peaks composing the broad emission structure, centred at 625 and 670 nm. **e**, Atomic force microscopy height images of **1a** (left) and **2** (right), deposited on highly oriented pyrolytic graphite.

Because of the bulky side groups, the *π*-stacking among **2** decreases significantly. MGNR **2** exhibits excellent solubility in dichloromethane, chloroform, toluene and so on just by mild shaking, rather than strong sonication, and the solutions are stable for months without observable precipitates. Concentrations (*C*) of 3.2 g l^–1^ in toluene and 5.0 g l^–1^ in chloroform are obtained, outperforming previously reported MGNRs. Suppression of *π*-stacking among carbon nanostructures is well-known to produce a bathochromic shift^18^, and **2** shows a UV–visible absorption peak that is blueshifted with respect to that of previously known MGNRs and **1b** (Fig. 2a). Excitons that are transferred inside the bundles quench the photoluminescence^19^, and indeed no luminescence is observed for MGNRs **1a** and **1b**; on the contrary, **2** exhibits bright photoluminescence in chloroform, confirming the suppression of aggregation (Fig. 2b). Below a concentration *C* = 0.1 g l^–1^, two photoluminescence peaks are observed between 600 and 650 nm, and the photoluminescence intensity increases with *C*, to saturate at ~0.1 g l^–1^ (Fig. 2c). Above this *C* value, the high-energy peak disappears and only the feature associated with inter-MGNR exciton transfer survives, with lower intensities on increasing *C*. The debundling is also observable after deposition on surfaces, for example, on highly oriented pyrolitic graphite, where atomic force microscopy reveals nano-sized stacks of MGNRs for **1a**, and single linear structures for **2** (Fig. 2d).

**Fig. 3:**
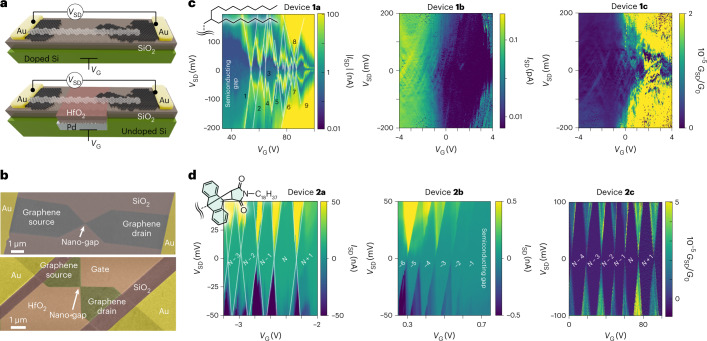
Enhancement of the quantum transport. **a**, Scheme of the device geometries used: a MGNR (side groups are not shown for clarity) bridges two graphene leads, connected to Au pads. The gate is either an n-doped Si wafer covered with a 300 nm SiO_2_ insulating layer (top) or a Pd electrode buried into undoped Si and covered with a 10 nm HfO_2_ layer (bottom). **b**, Scanning electron microscopy images of two typical devices for the two geometries, in false colours. **c**, Stability diagrams for three devices obtained with **1** (shown at the top left), showing the source–drain current *I*_SD_ versus bias, *V*_SD_, and gate, *V*_G_, voltages. **d**, Stability diagrams for three devices obtained with **2** (shown at the top left), showing the source–drain differential conductance *G*_SD_, in units of the conductance quantum *G*_0_. All measurements are taken below *T* = 500 mK, and, where possible, diamonds are labelled with respect to an arbitrary number of electrons, *N*.

We now proceed to observe the effect of debundling on the main element of quantum electronics, the single-electron transistor (SET). We tested different geometries in which the MGNRs span across a gap of width *d* = 1–10 nm electro-burned between two graphene electrodes. The gate is provided by either an n-doped Si wafer with a SiO_2_ layer with thickness *t*_ox_ ≈ 300 nm, or a Pd electrode etched into undoped Si and covered by 10 nm of HfO_2_ (Fig. 3a,b). A source–drain voltage *V*_SD_ and a gate voltage *V*_G_ can thus be applied while a source–drain current *I*_SD_ is measured (Fig. 3a and Supplementary Fig. 13)^12^. The MGNRs, which retain their covalently bonded side groups upon deposition, are added by drop casting from solutions, with a yield of roughly five devices out of a 100 electro-burned gaps, and the MGNR presence is detected as a sharp increase of the conductance *G* = ∂*I*_SD_/∂*V*_SD_ from <1 nS to ~100 nS (Supplementary Figs. 13–15).

In SETs, electron transport occurs via a single channel whose potential can be tuned, via *V*_G_, to bring the MGNR levels into resonance with the leads, so that electrons can tunnel into the MGNR. Because of Coulomb repulsion, an electron already present in the conduction channel hinders the passage of additional electrons, and SETs display regions of suppressed conductance. For **1**, bundling made it impossible to obtain SETs with only one MGNR bridging the nano-gap, and the typical stability diagram shows a vast region of suppressed conductance, corresponding to the bandgap of the semiconducting MGNRs, followed by multiple overlapping Coulomb diamonds with different sizes (Fig. 3c and Supplementary Fig. 14). While this is not the typical behaviour for a full carpet of MGNRs^20^, which would not produce observable diamonds, about a dozen nanoribbons are typically present in the nano-gaps. Because the MGNRs’ level spacing is smaller than the charging energy *E*_c_, the lengths *l* of the contributing nanoribbons can be roughly estimated from the addition energies *E*_a_ by considering them as rectangular-shaped quantum dots, for which $E_{\mathrm{a}} \approx E_{\mathrm{c}} = \frac{{e^2}}{{2C}} = \frac{{e^2{{{\mathrm{ln}}}}(4h/w)}}{{4\uppi \varepsilon _0\varepsilon _{\mathrm{r}}l}}$, where *e* is the electron charge, *h* is the ribbon–gate distance, *ε*_0_ is the dielectric constant and *ε*_r_ is the relative permittivity of SiO_2_ (ref. ^21^). Some of the most discernible diamonds yield *l* ≈ 28, 90, 40, 17, 108, 60, 50 and 33 nm, that is, values within the observed MGNR length distribution (Supplementary Fig. 8). No excited states are distinguishable inside the diamonds.

By stark contrast, SETs obtained with **2** display conduction features that are exceptionally clean and a level of detail comparable to that of ultra-clean CNTs (Fig. 3d and Supplementary Fig. 16). The diamonds are clearly periodic and display the same slopes and sizes, with clearly defined edges, indicating a single nanoribbon for each device. Discrete, faint lines inside the sequential-tunnelling regions that are slanted with respect to the diamond edges are produced by quantum interference effects in the graphene leads^22^. The MGNR excited states^1–4,15,16^ are clearly observable as intense, sharp lines that run parallel to the Coulomb edges. The high contact impedances observed are likely affected by contact lengths limited by the MGNR length, shorter than the transfer length, and by high Schottky barriers produced by the side groups, which can decrease the electrical contact between MGNR and the graphene leads.

**Fig. 4:**
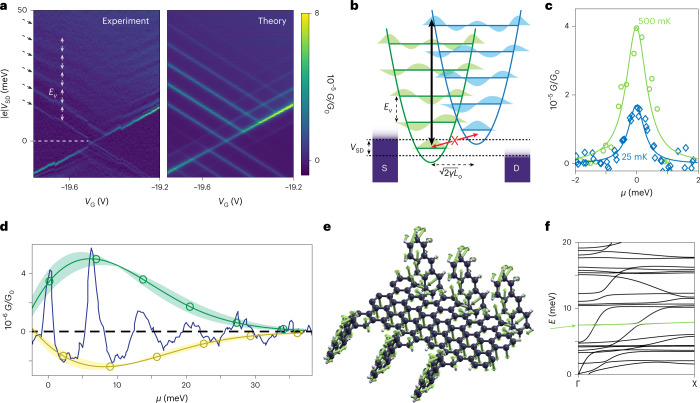
Electron–vibron coupling in nanoribbons with enhanced solubility. **a**, Detail of vibrational state suppression in the differential conductance *G* versus *V*_SD_ and *V*_G_ for **2** (left) and corresponding simulation using a quantum rate-equation model (right). Arrows indicate the excited states, and measurements are at *T* = 20 mK. **b**, Scheme of the Franck–Condon blockade in the transport properties of nanodevices, with the equilibrium coordinates represented horizontally for two adjacent charge states (green and blue). Strong vibronic coupling exponentially suppresses ground-to-ground-state transitions, while ground-to-excited-state ones become available at higher bias. S, source; D, drain. **c**, Lifting of the Franck–Condon blockade for a transmission channel upon increasing the temperature from 25 mK (blue) to 500 mK (green), together with Lorentzian fits to the data (lines). **d**, Blockade peaks as a function of the chemical potential *μ* as experimentally observed (blue line) and as expected by Franck–Condon theory for maxima (green) and minima (yellow). Shaded areas represent confidence intervals (as described in the main text). **e**, Relative displacement of the atoms for the 7 meV vibrational mode at the Γ point. **f**, Energy dispersion of the lowest vibrational modes, with the one at 7 meV highlighted in green.

A zoomed-in view into one of the diamonds highlights the exceptional cleanliness of the features and shows a full spectrum of the conducting element: the edges of the diamonds are suppressed, and a series of multiple excited states can be clearly observed (Fig. 4a and Supplementary Fig. 16), with a 7.0 ± 0.2 meV feature common to all devices. The suppression of the edge conductance is gradually lifted on increasing *T* (Fig. 4b). Similar to the Franck–Condon principle in molecular spectroscopy, electron–vibron coupling will suppress transport for low-lying vibronic states, because electrons are most likely to be transmitted when changes in the positions of the nuclei are minimal (Fig. 4c). For vibrons in equilibrium, the peak conductances $G_{n,0}^{{\mathrm{max}}}$ associated with the Franck–Condon transition probabilities follow the progression $G_{n,0}^{{\mathrm{max}}} = \frac{{\gamma ^n}}{{n!}}{e}^{ - \gamma }$, where *γ* affords the strength of the electron–vibron coupling and *n* is the vibron quantum number, and excellent agreement with the data is observed for the whole Franck–Condon progression of excited vibrational states, yielding *γ* = 1.5 ± 0.2 (Fig. 4d and Supplementary Figs. 18–20). In CNTs, suspension is necessary to achieve large *γ* values (*γ* = 3.3)^23^ because low-energy modes in the substrate phonon bath assist sequential-tunnelling conduction at the diamond edges and quench the electron–vibron coupling. MGNRs here reach values higher than that of unsuspended nanotube junctions (0.5 < *γ* < 1.1)^24^, which validates theoretical predictions of superior electron–vibron coupling^25^ and also hints at partial substrate decoupling by the flexible edge groups.

The observed vibrational frequency *ω*_*v*_ and the associated quantization energy *E*_*v*_ = *ħω*_*v*_ (where *ħ* is the reduced Planck’s constant) are much higher than the catenan modes of a 300-nm-long vibrating CNT, indicating that the MGNR electronic states couple to very different vibrational modes. The electronic states of the MGNR are largely edge localized^26^ and can overlap and couple with the edge vibrational modes. Because the edge structure of the MGNR is perfectly defined, we can calculate the vibrational properties. The transverse bending mode, which involves the displacement of the edge atoms with respect to the backbone, indeed appears at *ħω*_*v*_ = 7 meV, in excellent agreement with the experiments (Fig. 4e,f). Removal of the bulky side groups lowers the mode energy slightly to 5.4 meV, with a larger displacement of the edge atoms, without altering the effect on the MGNR backbone (Supplementary Fig. 17). All vibrons at frequencies *ω*_*v*_ and the associated electron–vibration coupling strengths *g*_*v*_ can be included in a quantum-mechanical description of the electron transfer rates through the MGNR density of states^27^:

$$K_{\mathrm{e}} = \frac{1}{\uppi }{{{\mathrm{Re}}}}\mathop {\smallint }\limits_0^\infty {\mathrm{e}}^{i\left( {E - \mu } \right)t/\hbar }{\mathrm{e}}^{ - t/\tau }\xi (t){\mathrm{d}}t$$

where *τ* is the line broadening associated with the electronic state lifetime, *μ* is the MGNR energy level, *t* is time, *i* is the imaginary unit, *E* is energy and *ξ*(*t*) describes the nuclear dynamics associated with electron transfers, as modulated by a time-dependent Franck–Condon factor $\xi (t) = \exp \left\{ {{\int} {\frac{{J(\omega )}}{{\omega ^2}}} \left[ {\left( {\cos \omega t - 1} \right)\coth \left( {\frac{\omega }{{2k_{\mathrm{B}}T}}} \right) - i\sin \omega t} \right]{\mathrm{d}}\omega } \right\}$ where *ω* is the frequency and *k*_B_ is the bolzmann factor and where the vibrational spectral density is $J\left( \omega \right) = \mathop {\sum }\limits_v g_v^2\updelta (\omega - \omega _v)$ for the MGNR modes. The outer-sphere background, produced by the weak coupling of the MGNR with the substrate, can also be included by adding a super-ohmic spectral density with an exponential cut-off (Supplementary Information)^22^. Excellent agreement is obtained with the data, and the entire current map can be faithfully reproduced (Fig. 4a).

These results demonstrate electronic devices of extreme cleanliness made out of MGNRs, with sharply defined electronic features that are much superior to those of previous observations. Chemically, this opens a whole area of synthetic design aimed at placing different and increasingly efficient solubilizing groups on the edges. The resulting regularly spaced structural elements allow one to avoid the disorder associated with surfactants and the decrease in performance that has plagued CNTs. The resulting debundled solution is simply a two-component system without surfactants and other additives. For nanoelectronics, this result reveals completely new perspectives: ultra-clean details are now available for graphene nanodevices that have atomically defined shapes. For the vibrational properties, this allows one to establish a direct correspondence between the vibrational modes of the specific edge structure and the observed electronic features. Remarkably, it is not necessary to suspend the nanoribbons to clearly observe the physics of vibrational states, and the resulting electron–vibron coupling is comparable to that of ultra-clean CNT devices, validating the prediction of superior electron–vibron coupling^23^ and opening the path to the envisaged nanomechanical devices based on atomically precise nanoribbons^28^. Strategies towards higher conductance by tuning the contact resistance or increasing mobility can now be explored, for example by using wider MGNRs or by introducing doping with electron-rich side groups; and chemical preselection of the MGNR lengths, for example, via size-exclusion chromatography, opens up the path to low-impedance contacts. As nanoribbon states are now resolved in transport at microelectronvolt energies, the predicted spin and topological states can now be accessed with remarkable simplicity, and their manipulation will soon become a reality, together with the exploitation of the long edge-spin coherence times.

## Methods

### Synthesis

All the reagents were purchased from Sigma-Aldrich, TCI, Fluorochem and other commercial suppliers. All reactions dealing with air- or moisture-sensitive compounds were carried out in a dry reaction vessel under argon. A degassed solution of **2a** (450 mg, 0.59 mmol, synthetized as reported in the Supplementary Information) in diphenyl-ether (0.40 ml, 1.5 M) was refluxed for 20 h, and CH_3_OH was added at room temperature. The resulting crude polymer collected by filtration was fractionated by gel permeation chromatography recycling, leading to **2b**. The **2b** (100 mg) and *N*-octadecylmaleimide (0.95 g, 20 equiv. for each repeating unit) were added to anhydrous *o*-xylene (10 ml) and degassed. The mixture was stirred at 150 °C for 72 h, and CH_3_OH was added at room temperature. Gel permeation chromatography recycling was used to remove the excess *N*-octadecylmaleimide, yielding **2c** (120 mg, 89% yield, 77% grafting ratio). A suspension of FeCl_3_ in nitromethane (515 mg for 5 ml) was added to a solution of **2c** (50 mg) in anhydrous and degassed dichloromethane (50 ml). After stirring at room temperature for 72 h under Ar bubbling, the reaction was quenched by the addition of CH_3_OH. The title compound (dark-red powder, 47 mg, 95% yield) was obtained by filtration followed by washing with CH_3_OH and water.

### Optical measurements

UV–visible spectra were measured with an Agilent Cary 5000 spectrophotometer. Fluorescence spectra were recorded on a PerkinElmer Fluorescence Spectrometer LS55.

### Surface measurements

Atomic force microscopy measurements were performed on freshly exfoliated highly oriented pyrolitic graphite using Bruker Dimension Icon and Asylum Research Cypher-S atomic force microscopes in tapping mode.

### Electronic devices

Chemical vapor deposition graphene was transferred onto wafers with prepatterned Cr–Al (10–70 nm) electrodes using wet-transfer techniques and etched into bow-tie shapes using electron-beam lithography followed by O_2_-plasma etching. A gap was opened in the graphene constriction using feedback-controlled electro-burning in air, and *I*_SD_–*V*_SD_ characteristics were recorded for every device. MGNR dispersions of **1** and **2** were prepared by sonication in tetrahydrofuran for 10 min; ~8 μl were drop cast onto the nano-gaps immediately after electro-burning the devices and measured. Transport measurements were performed in an OI-HelioxVT ^3^He cryostat and an OI-Triton dilution refrigerator using low-noise d.c. electronics (Delft and homemade).

### Simulations

For the density functional theory calculations, we used Siesta using the local-density approximations functional with an energy cut-off of 800 Ry, and the Monkhorst–Pack *k*-point grid was (50, 1, 1). The structure was optimized until the maximum force on the atoms was less than 0.004 eV Å^–1^. The dispersion relation was calculated with a supercell size of (3, 1, 1), and the atomic displacement was visualized with XCrySDen.

## Online content

Any methods, additional references, Nature Portfolio reporting summaries, source data, extended data, supplementary information, acknowledgements, peer review information; details of author contributions and competing interests; and statements of data and code availability are available at 10.1038/s41563-022-01460-6.

## Supplementary information

Supplementary Information Supplementary Figs. 1–20, Table 1 and Discussion.

## Data Availability

The data supporting the findings of this study are available within the Article and its Supplementary Information, and are deposited and available online within the Bodleian Library of Oxford. No custom code is used.
