# Supplementary material for: Exceptionally clean single-electron transistors from solutions of molecular graphene nanoribbons
Source: Nat Mater. 2023 Feb 2;22(2):180–5. doi: 10.1038/s41563-022-01460-6 (PMC10208969; doi:10.1038/s41563-022-01460-6)
Supplement: Supplementary file 1 — Supplementary Figs. 1–20, Table 1 and Discussion. [file 41563_2022_1460_MOESM1_ESM.pdf]

nature materials

Letter

https://doi.org/10.1038/s41563-022-01460-6

Exceptionally clean single-electron transistors from solutions of molecular graphene nanoribbons

In the format provided by the authors and unedited

Supplementary Information for: Exceptionally-clean Single-Electron-Transistors from Solutions of Molecular Graphene Nanoribbons

Wenhui Niu,[1,2,†] Simen Sopp,[3,†] Alessandro Lodi,[3,†] Alex Gee,[3,†] Fanmiao Kong,[3] Tian Pei,[3] Pascal Gehring,[3] Jonathan Nägele,[4] Chit Siong Lau,[3,‡] Ji Ma,[1] Junzhi Liu,[1] Akimitsu Narita,[5] Jan Mol,[3,§] Marko Burghard,[4] Klaus Müllen,[5] Yiyong Mai,[2] Xinliang Feng[1] and Lapo Bogani[3,]*

[1] Center for Advancing Electronics Dresden, Faculty of Chemistry and Food Chemistry, Technische Universität Dresden, Mommsenstraße 4, 01069 Dresden, Germany

[2] School of Chemistry and Chemical Engineering, Shanghai Jiao Tong University, 800 Dongchuan RD, Shanghai 200240, China

[3] Department of Materials, University of Oxford, 16 Parks Road, OX1 3PH, Oxford, United Kingdom.

[4] Max Planck Institut für Festkörperforschung, Heisenbergstraße 1, 70569, Stuttgart, Germany.

[5] Max Planck Institut für Polymerforschung, Ackermannweg 10, 55128, Mainz, Germany.

[†] *These authors contributed equally to this work.*

[‡] *Current address: Institute of Materials Research and Engineering, 2 Fusionopolis Way, Innovis, 08-03, 138634 Singapore.*

[§] *Current address: School of Physics and Astronomy, Queen Mary University of London, London, UK.*

General Methods

NMR spectra were recorded on Bruker spectrometer (300 or 600 MHz) and CD_2Cl_2 ($\delta(^1H)$ = 5.33 ppm, $\delta(^{13}C)$ = 53.7 ppm) was used as solvent, lock and internal standard. The mass spectrometry analysis was performed on a Bruker Autoflex Speed MALDI TOF MS (Bruker Daltonics, Bremen, Germany) using *trans*-2-[3-(4-*tert*-butylphenyl)-2-methyl-2-propenylidene]malononitrile as matrix. Relative molar masses were determined by gel permeation chromatography (GPC) with an Aligent Technologies 1260 Infinity LC system equipped with two Resipore columns and RI and UV-vis detection. Chloroform was used as eluent with a flow rate of 1 mL min^{-1}. The measurements were carried out at 40 °C and the molar masses were calculated relative to polystyrene standards with low dispersity. Recycling gel permeation chromatography (rGPC) was performed on JAI HPLC LC 9110 II NEXT with fraction collector FC-3310 and GPC columns 2H and 1H (connected in series), the rGPC was used with HPLC-grade chloroform at room temperature. Raman spectra were recorded at ambient temperature using a Bruker Vertex 70 instrument equipped with a RAM II module (Nd-YAG laser, 1064 nm). Thermal gravimetric analyses (TGA) were carried out on Waters TGA Q500 by heating the samples from 25 to 1000 °C under Argon atmosphere at a heating rate of 10 °C min^{-1}. Fourier transform infrared (FT-IR) spectra were recorded on a Bruker Optics ALPHA-E spectrometer with a universal Zn-Se ATR (attenuated total reflection) accessory in the 600~4000 cm^{-1}.

Synthesis

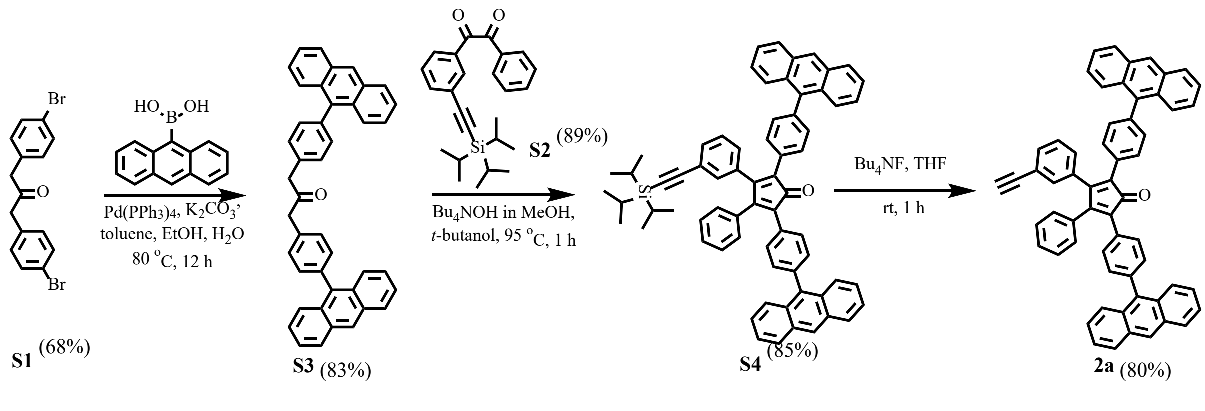

Figure S1: Synthetic route to **2a**.

The starting materials **S1**, **S2**, **S5** were synthesized adapting published protocols.[1,2]

1,3-Bis(4-(anthracen-9-yl)phenyl)propan-2-one (S3). In a 100 mL Schleck flask, compound **S1** (1.00 g, 2.72 mmol), 9-anthraceneboronic acid (1.81 g, 8.15 mmol) and K_2CO_3

(1.87 g, 13.60 mmol) were dissolved in 30 mL of toluene, 7 mL ethanol, and 7 mL water, then degassed by Ar bubbling for 1h. $Pd(PPh_3)_4$ (314 mg, 0.27 mmol) was added into the solution. Then the reaction was stirred at 80 °C for 12 h. Afterwards, the reaction mixture was diluted with DCM (50 mL) and filtered. The mixture was washed three times with water, dried over sodium sulfate, and evaporated. The solid was purified by silica column chromatography (eluent: hexane/DCM = 4/1) to give **S3** as a yellow solid (1.27 g, 83% yield). ^1H NMR (300 MHz, CD_2Cl_2) δ 8.52 (s, 2H), 8.06 (d, 8.4 Hz, 4H), 7.70 (d, 8.7 Hz, 4H), 7.55-7.40 (m, 12H), 7.39-7.29 (m, 4H), 4.05 (s, 4H) (Fig.S2). ^{13}C NMR (75 MHz, CD_2Cl_2) δ 205.96, 137.97, 137.16, 134.20, 132.07, 131.96, 130.76, 130.30, 128.86, 127.22, 127.10, 125.97, 125.70, 49.74 (Fig.S3). HRMS (MALDI-TOF) m/z: $[M]^+$ Calcd for $C_{43}H_{30}O$ 562.2297; found, 562.2297.

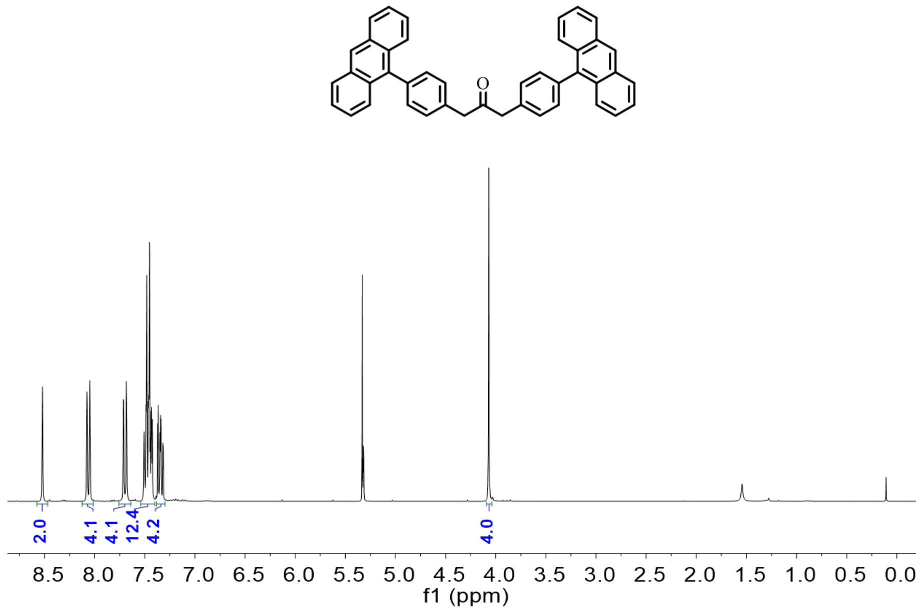

Figure S2: ^1H-NMR (300 MHz, CD_2Cl_2) spectrum of **S3**.

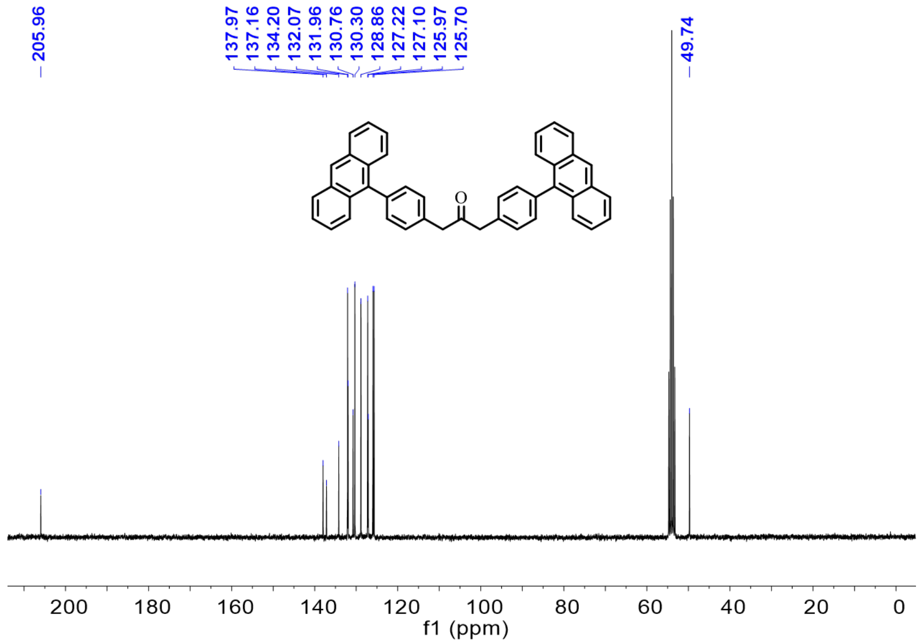

Figure S3: ^{13}C-NMR (75 MHz, CD_2Cl_2) spectrum of **S3**.

2,5-Bis(4-(anthracen-9-yl)phenyl)-3-phenyl-4-(3((triisopropylsilyl)ethynyl)phenyl) cyclopenta-2,4-dien-1-one (S4). To a solution of **S2** (387 mg, 0.97 mmol) and **S3** (546 mg, 0.97 mmol) in *t*-butanol (30 mL) was added a solution of tetrabutylammonium hydroxide (1 M in methanol, 0.49 mL). After stirring at 95 °C for 1 h, the reaction was quenched by the addition of 1 N HCl (20 mL), and the reaction mixture was extracted three times with dichloromethane. The combined organic layers were washed twice with water, dried over sodium sulfate, and evaporated to give a purple crude product. Purification by silica gel column chromatography (eluent: hexane/DCM = 3/1) gave **S4** as a purple solid (756 mg, 85% yield). ^{1}H NMR (600 MHz, CD_2Cl_2) δ 8.52 (s, 2H), 8.07 (d, 8.5 Hz, 4H), 7.71 (d, 8.4 Hz, 4H), 7.58-7.52 (m, 4H), 7.50-7.47 (m, 4H), 7.43-7.30 (m, 12H), 7.27 (t, 7.7 Hz, 1H), 7.21-7.14 (m, 4H), 1.11-1.07 (m, 21H) (Fig.S4). ^{13}C NMR (150 MHz, CD_2Cl_2) δ 201.09, 155.87, 154.62, 138.70, 138.54, 137.19, 137.14, 134.02, 133.70, 133.52, 132.41, 131.96, 131.67, 131.56, 130.77, 130.69, 130.67, 130.65, 130.56, 130.02, 129.92, 129.40, 128.86, 128.76, 127.24, 127.23, 127.16, 127.14, 126.12, 126.04, 126.00, 125.78, 125.72, 125.71, 123.97, 106.92, 91.92, 78.11, 18.98, 11.85 (Fig.S5). HRMS (MALDI-TOF) m/z: $[M]^+$ Calcd for $C_{68}H_{56}OSi$ 916.4100; found, 916.4092.

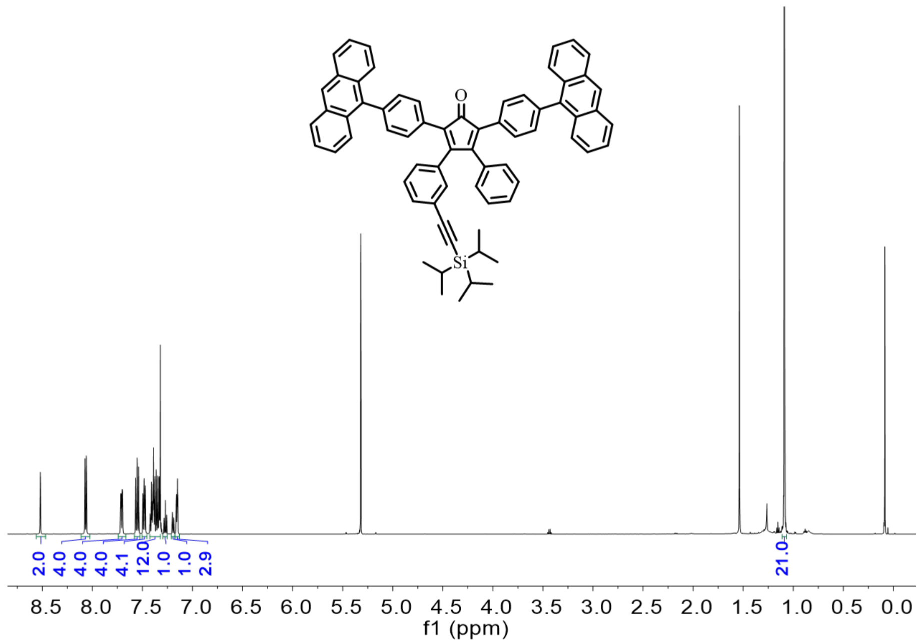

Figure S4: ^{1}H-NMR spectrum (600 MHz, CD_2Cl_2) of **S4**.

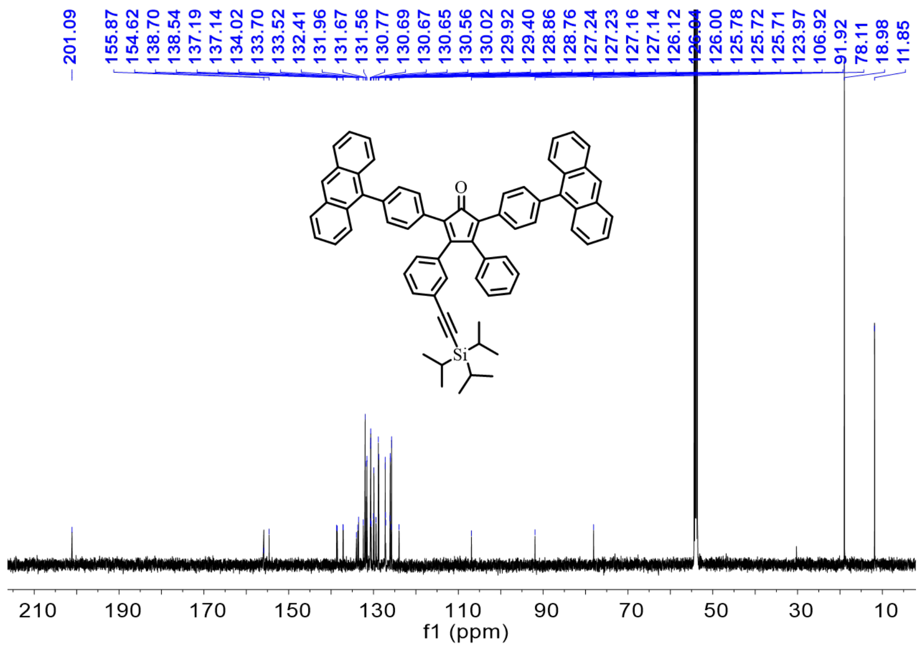

Figure S5: ^{13}C-NMR spectrum (150 MHz, CD_2Cl_2) of **S4**.

2,5-Bis(4-(anthracen-9-yl)phenyl)-3-(3-ethynylphenyl)-4-phenylcyclopenta-2,4-dien-1-one (2a). To a solution of **S4** (0.90 g, 0.98 mmol) in anhydrous THF (100 mL) was added a solution of tetra-*n*-butylammonium fluoride (1 M in THF, 1 mL) dropwise over 5 min. After stirring at room temperature for 1 h, methanol was added to the reaction mixture and solvent

was removed in vacuo at 40 °C. Purification by silica gel column chromatography (eluent: hexane/DCM = 3/1) yielded compound **2a** as a purple solid (597 mg, 80% yield). ^{1}H NMR (600 MHz, CD_2Cl_2) δ 8.53 (s, 2H), 8.07 (d, 8.5 Hz, 4H), 7.73 (dd, 9.0, 4.7 Hz, 4H), 7.59-7.47 (m, 8H), 7.46 (d, 7.7 Hz, 1H), 7.44 -7.30 (m, 12H), 7.26 (t, 7.8 Hz, 1H), 7.18-7.15 (m, 2H), 7.07 (dt, 7.8, 1.2 Hz, 1H), 3.15 (s, 1H) (Fig.S6). ^{13}C NMR (150 MHz, CD_2Cl_2) δ 200.91, 155.43, 154.59, 138.78, 138.59, 137.17, 137.12, 134.14, 133.62, 133.58, 132.76, 131.96, 131.67, 131.58, 130.71, 130.68, 130.66, 130.53, 130.51, 129.95, 129.40, 128.87, 128.86, 128.79, 127.25, 127.24, 127.17, 127.16, 126.66, 126.05, 126.02, 125.89, 125.73, 122.68, 83.39, 78.14 (Fig.S7). HRMS (MALDI-TOF) m/z: $[M]^+$ Calcd for $C_{59}H_{36}O$ 760.2766; found, 760.2748.

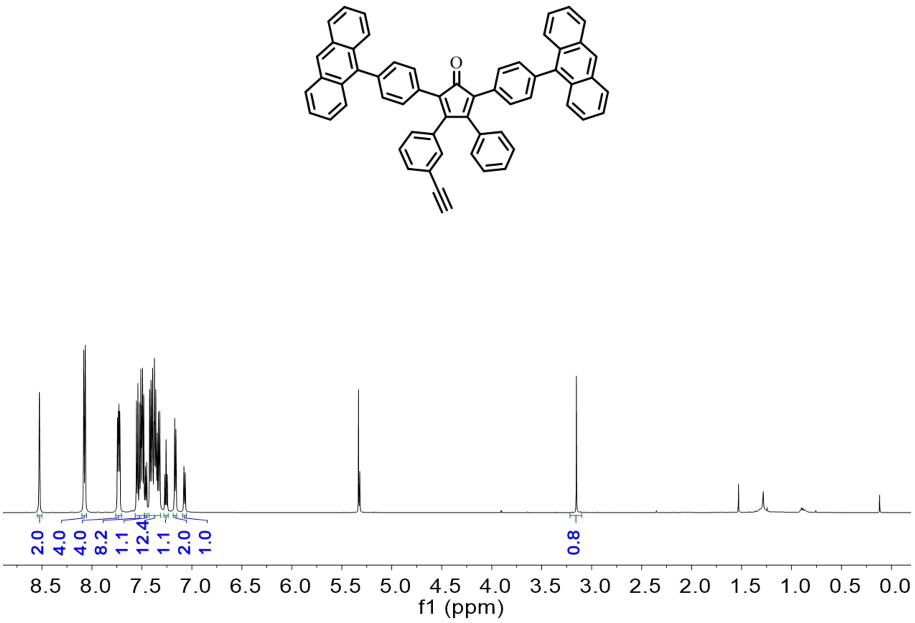

Figure S6: ^{1}H-NMR spectrum (600 MHz, CD_2Cl_2) of **2a**.

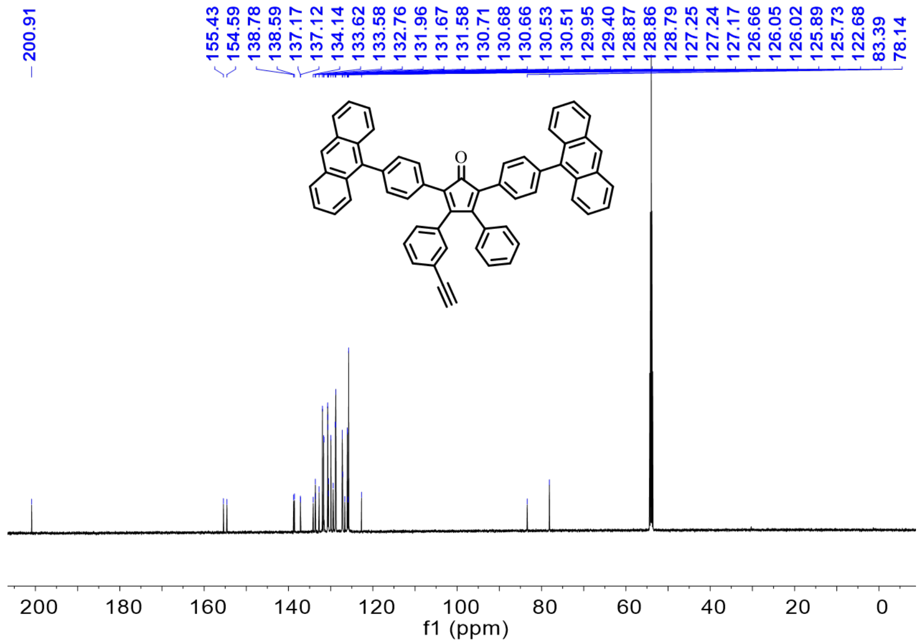

Figure S7: ^{13}C-NMR spectrum (150 MHz, CD_2Cl_2) of **2a**.

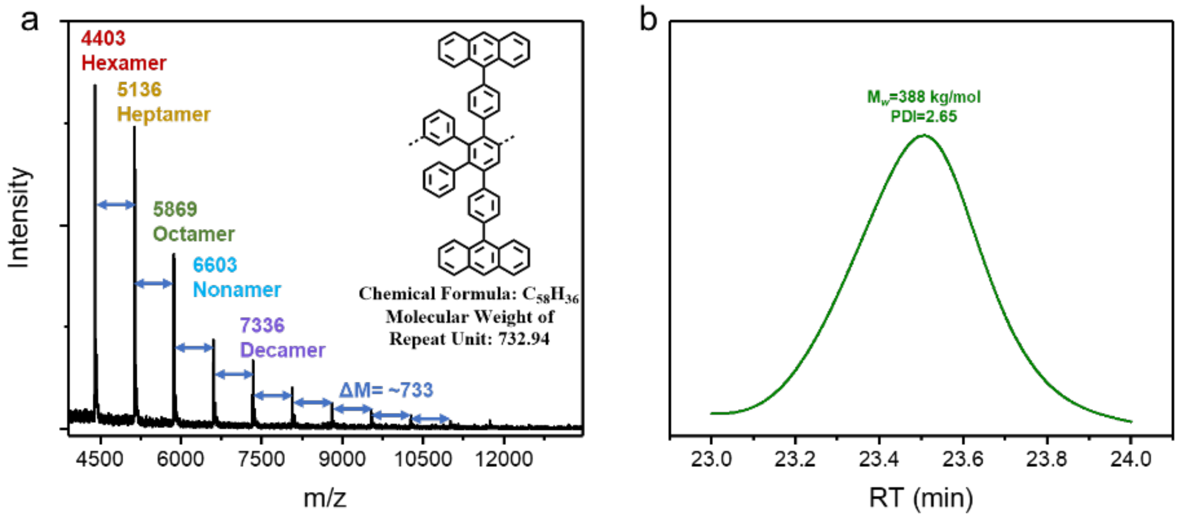

Figure S8: (a) MALDI-TOF MS characterization and (b) GPC curve of **2b**. The length of the resultant MGNR **2** was calculated to be 371 nm according to the M_w value of **2b**.

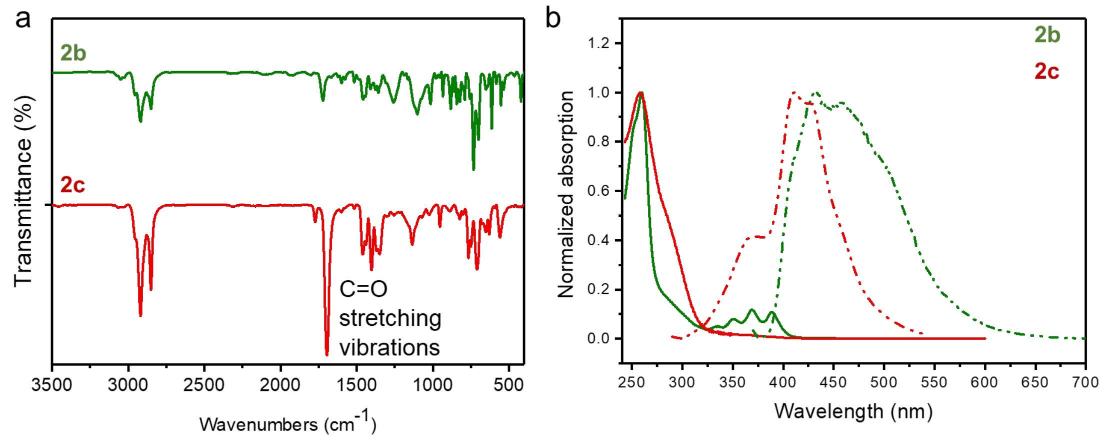

Figure S9: (a) The FTIR spectra of **2b** and **2c**. (b) UV-vis spectra (solid lines) and fluorescence spectra (dashed lines) spectra of **2b** and **2c**.

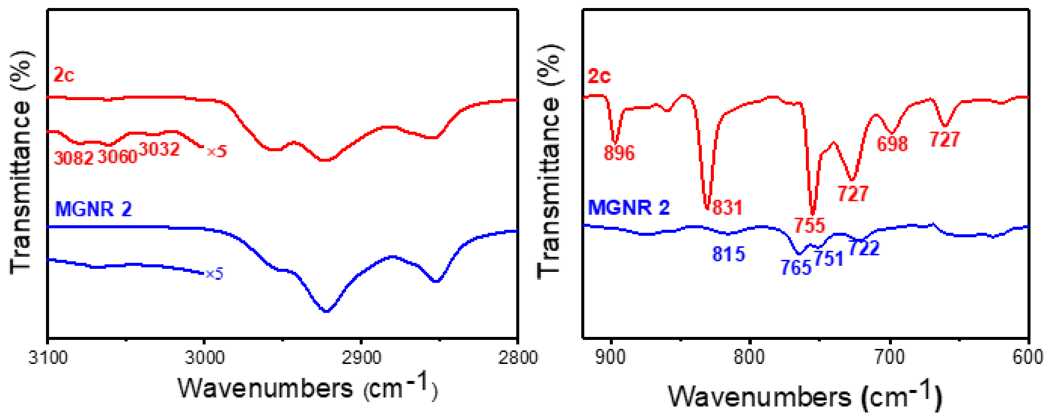

Figure S10: Representative FTIR spectral regions of **2c** (red lines) and pleio-soluble MGNR **2** (blue lines) show disappearance of the bands derived from mono- and disubstituted benzene rings on graphitization.

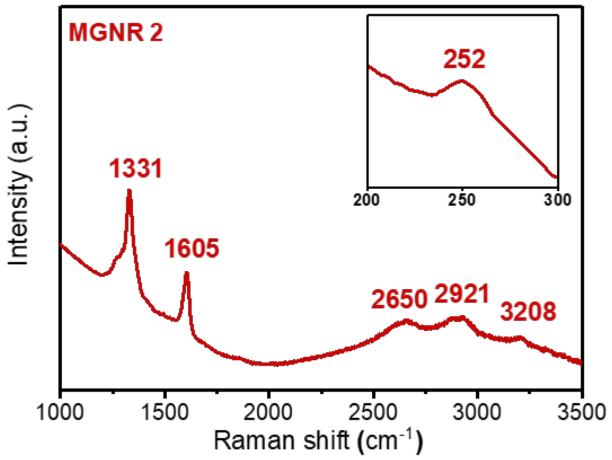

Figure S11: Raman spectrum of pleio-soluble MGNR **2** measured at 532 nm. The inset displays the RBLM peak at 252 cm^{-1}.

The grafting ratio (GP) of the bulky side-groups in MGNR **2** can be calculated according to thermal gravimetric analyses (TGA):

$$w_{\mathrm{maleimide}} = 1 - w_{\mathrm{residue}}\left(1 + \frac{2m_{\mathrm{anthracene}}}{m_{\mathrm{backbone}}}\right)$$

$$= 1 - 0.29\left(1 + \frac{177 \cdot 2}{364}\right) = 0.43.$$

$$\mathrm{GP} = \frac{w_{\mathrm{maleimide}}/m_{\mathrm{maleimide}}}{2w_{\mathrm{residue}}/m_{\mathrm{backbone}}} = \frac{0.43/349}{2 \cdot 0.29/364} = 77\%,$$

where w_{residue} is the weight fraction of the GNR rigid backbone, which still retained after thermal treatment over 500 °C according to the TGA curve; 364, 177 and 349 represent the molar mass, m, of GNR backbone, anthracene and maleimide, respectively.

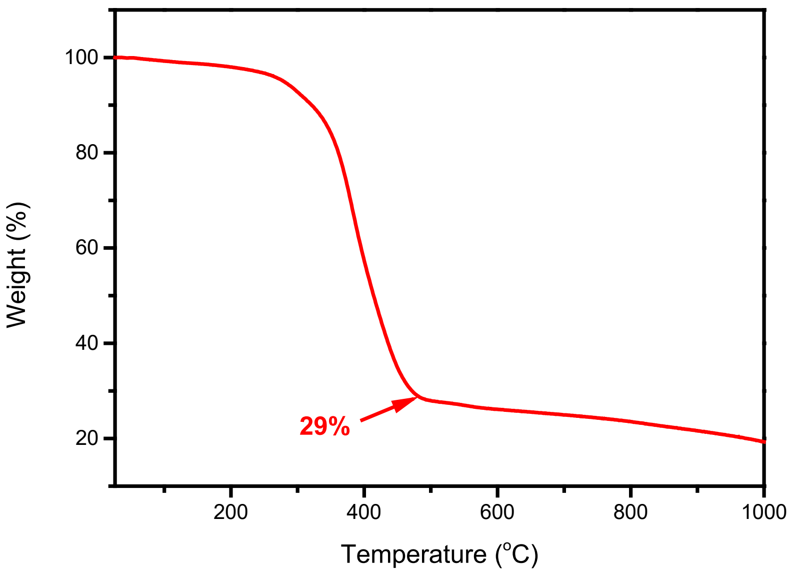

Figure S12: TGA curve of pleio-soluble MGNR **2**.

Fabrication Procedure of 1b

Each device is made of doped Si (nominal sheet resistance of 0.001 Ω/cm^2) as back-gate and 300 nm of SiO_2 thermally growth on top of it as insulating material. A single chip (1 x 1 cm^2) hosts 540 pairs of Cr (10 nm) / Au (70 nm) patterned by EBL lithography and metal evaporation. CVD graphene was prepared by using a 1% CH_4:Ar gas mixture on liquid copper at atmospheric pressure and a temperature of 1090 °C. After transferring CVD graphene/PMMA stack on top with wet transferring method, 200 nm wide notched ribbon were printed on each device followed by an oxygen plasma etching to remove unexposed graphene. Before the electroburning, the chips were annealed at 350 °C in an argon atmosphere for one hour to further clean the graphene surface.

Transport Properties of 1b

Room Temperature. The device using **1b** was fabricated on a chip of patterned graphene electroburnt to open a nanogap separating the graphene leads. The I–V_{SD} characteristics of the junction before and after deposition of **1b** are shown in Figure S13a. Pre-deposition the

transport is dominated by electrode-electrode tunnelling. The sharp increase in conductivity after deposition indicates that the nanogap is bridged by MGNRs.

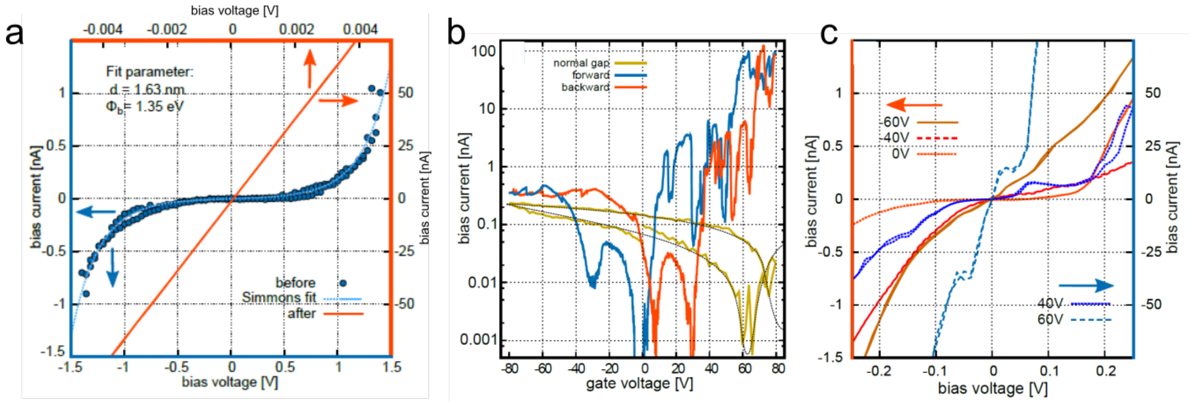

Figure S13: (a) I–V_{SD} characteristics of the junction before (blue dots; bottom horizontal axis and left vertical axis) and after (red line; top horizontal axis and right vertical axis) deposition of MGNRs (**1b**). A Simmon's fit (see text) of the empty junction is plotted in a dotted blue line. (b) V_g current traces at V_{sd}=0.1 V for a graphene device before electroburning (yellow line) and for the MGNR-carpet junction (blue and red lines) at T = 7 K. (c) V_{sd} current traces at various V_g at T=7 K.

Low Temperature. Transport spectroscopy at T = 7 K was performed and revealed strong current asymmetry both in V_g traces (Fig.S13b) and V_{sd} traces (Fig.S13c). Figure S13b shows V_g traces of a graphene device (before nanogap creation) and a device where several MGNRs bridges the gap. The resistance-limited current through the graphene device reaches a minimum around $V_g = 60$ V, which likely corresponds to the Dirac point. The current through the MGNR device is considerably higher and depends on the position of the Fermi level. Significant hysteresis is present in both devices and can be attributed to trapped charges nearby, e.g. in the substrate. Figure S13c shows V_{sd} traces with clear current-steps as is typical for sequential electron tunnelling.

Stability diagrams of the device showed Coulomb diamonds of many different sizes, which indicate the presence of multiple transport channels with different addition energies (from different MGNR lengths) and electrode coupling strengths (Figs.S15a,b,d). The red and the black diamond patterns in Figures S15a and S15b highlight Coulomb diamonds originating from two different MGNRs (among many) that bridges the nanogap. The gate lever arm, α, for these two sets were estimated from the slopes of the diamonds as α=0.015 and α=0.007 for the

red and the black sets, respectively. Their respective addition energies, E_{add}=150 meV and E_{add}=70 meV were extracted from the height of the diamonds. Figure S15c highlights the evolution of the I–V_{SD} characteristics as a function of V_{G} close to a degeneracy point.

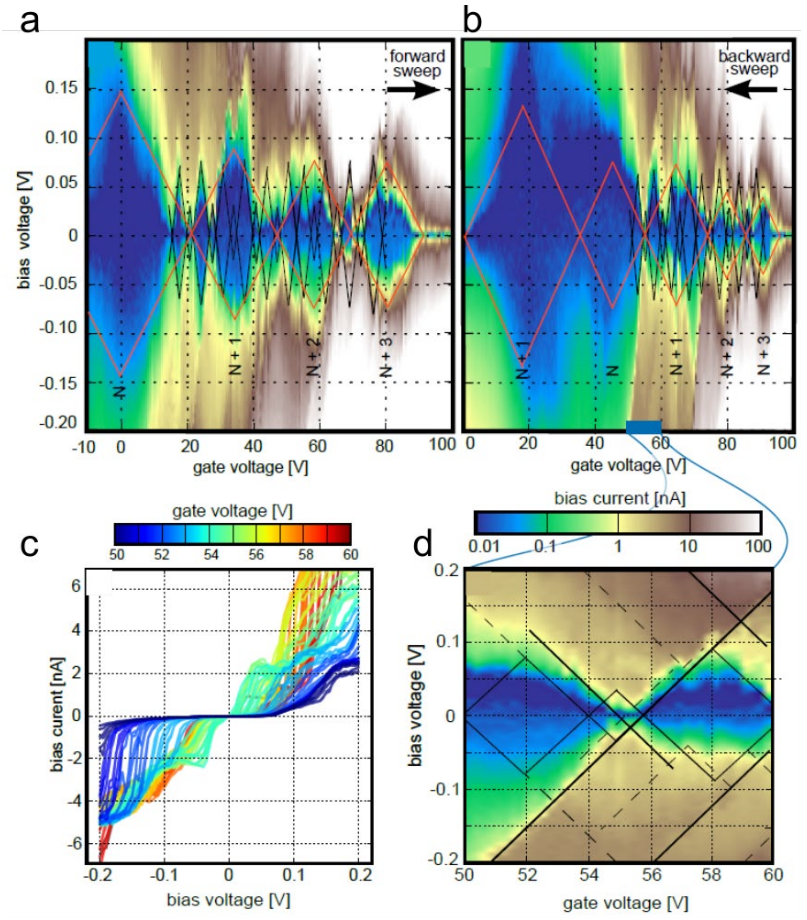

Figure S14: Stability diagrams of device with **1b** acquired at T=7 K after sweeping V_g from (a) negative to positive and from (b) positive to negative. Overlays of black and red diamond patterns indicate signatures of conduction channels from multiple MGNRs. (c) V_{sd} current traces acquired at V_g from 50 to 60 V. (d) A section of (b), in the same V_g range as in (c), with overlays indicating different Coulomb diamond edges.

Fabrication Procedure of 2

Each device is made of doped Si (nominal sheet resistance of 0.001 Ω/cm^2) as back-gate and an insulating layer of 300 nm of SiO_2 thermally grown on top of it. A single chip (1 x 1 cm^2) hosts 874 pairs of Cr (10 nm)/Au (70 nm) patterned by EBL lithography and metal evaporation. The whole wafer was then sent to Graphenea for wafer-scale transferring of CVD monolayer graphene, with 600 nm of PMMA stacked on top of it. PMMA was then removed by leaving

the chip in warm acetone overnight. Negative resist ARN-7500 was spun at 4000 rpm for 60 s, baked at 85 °C and exposed in EBL at 300 μC/cm^2. 200 nm-wide graphene notches were then developed in MF-CD-26 for 60 s, immersed in deionised water for 60 s (no agitation), and blow-dried with N_2.

Unwanted graphene features were removed by etching with a Henniker Oxygen Plasma (25 scmm, 50 % of power for 90 s). Afterwards, chips were immersed in REM-660 for one hour to remove cross-linked resist (agitation is not needed). The chips were rinsed in acetone for 1 min, followed by 1 min in IPA, and gently blow-dried with N_2. Finally, nanogaps were formed using an electro-burning protocol and ~8 μL of a 1 mg/mL solution of **2** was drop casted onto the chip immediately after the formation of the junction.

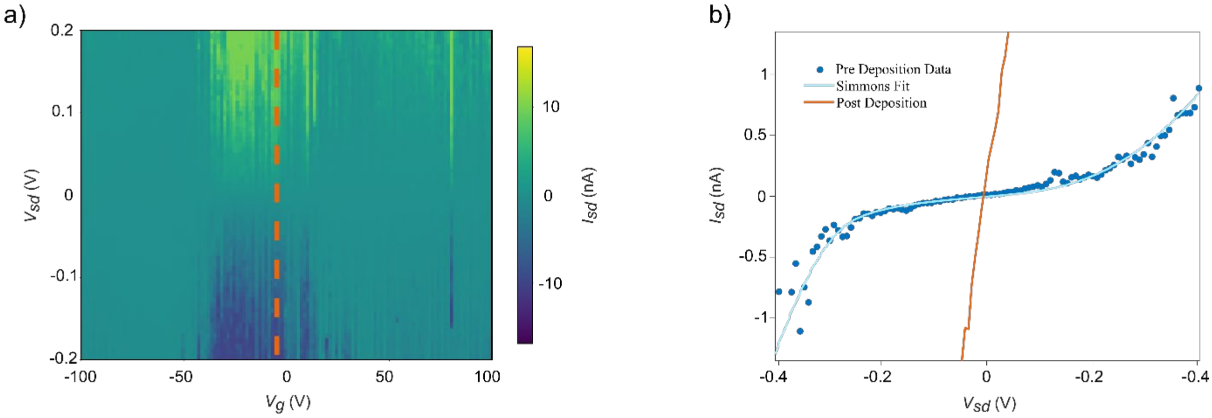

Figure S15: Room-temperature transport properties of a device with **2** acquired in air before and after drop casting. (a) Stability diagram of a device after drop casting. The vertical orange line identifies the gate voltage where we extracted the bias trace plotted in (b). (b) I–V_{SD} traces after electro-burning of a nanogap (blue filled dots) and after the deposition of **2** on the device (orange solid line). The tunnelling current yielded by the electro-burning protocol has been fit with the Simmons model (blue line).

Room-temperature Transport Properties of 2

Figure S16a shows the stability diagram for a device where **2** has been successfully deposited through drop casting, taken at room temperature in air. Figure S16b shows the tunnelling current of the break–junction after the electro-burning, the Simmons fit[3] (fitting paramaters: ϕ_{avg} = 0.47 eV, pre-factor = 0.62 nA, gap-size = 1.28 nm), and the current level after solution containing **2** has been deposited on top of the device. The observed increase in conductance by several orders of magnitude after deposition indicates that drop casting has resulted in one

MGNR randomly bridging the nanogap. In the main text we discuss that there is only one MGNR because there is only one set of Coulomb diamonds in the stability diagrams at low *T*.

Low-temperature Transport Properties of 2

Identifying Conductance Peaks of Vibrational Origin. Sequential electron tunnelling (SET) events may be assisted or suppressed by molecular vibrational states in accordance with the Franck-Condon principle[4]. An analysis of such phenomenon first requires the identification of the vibrational states of the MGNR. Resonant SET manifests as conductance peaks in V_{sd} traces and as conductance ridges in the stability diagram(e.g. as in Fig. 4a in the main text). The chemical potential, $\mu = |e|V_{sd}$, of states in resonance with the leads can be read off the V_{sd} axis.

The conducting regions of the stability diagrams may contain resonant ground- and excited states of several origins: electronic, vibrational, spin, and lead. A prerequisite for Franck-Condon analysis is to isolate the vibrational states. By following the harmonic oscillator model, vibrational states are typically evenly spaced in energy, a spacing that does not change significantly for different charge states, does not depend on a magnetic field, and run parallel to the diamond edges. Electronic states often change significantly for different charge states and depends on magnetic fields. Conductance peaks originating from density-of-states fluctuations of the graphene leads are often characterised by running non-parallel to the diamond edges and exhibiting a periodical pattern of positive and negative differential conductance. Therefore, looking for evenly spaced conductance peaks that run parallel to the diamond edge, and do not shift or split in a magnetic field, is one method of isolating vibrational states from the others. Caution is advised when using this method because not all electronic states (e.g. singlets) will split in a magnetic field and may be mistaken for a vibrational state. Ab-initio calculations of the vibrational states should be used in combination to verify or suggest vibrational modes.

Visual inspection of the stability diagram in Figure 3d in the main text revealed conductance ridges evenly spaced by ~7.5 meV in all conducting regions that did not split with magnetic field. To differentiate between slanted conductance ridges from the leads, a script was made to average the conductance along lines running parallel to the diamond edges. In this way, only conductance ridges running approximately parallel to the diamond edges remained while slanted ridges were eliminated. The assumption is thus that the average conductance as a function of $|e|V_{sd}$ excludes the contribution of the lead transitions. The second assumption is

that only peaks separated by ~7.5 meV represent the vibrational states of interest (thereby excluding electronic states). Figure S13a shows this procedure.

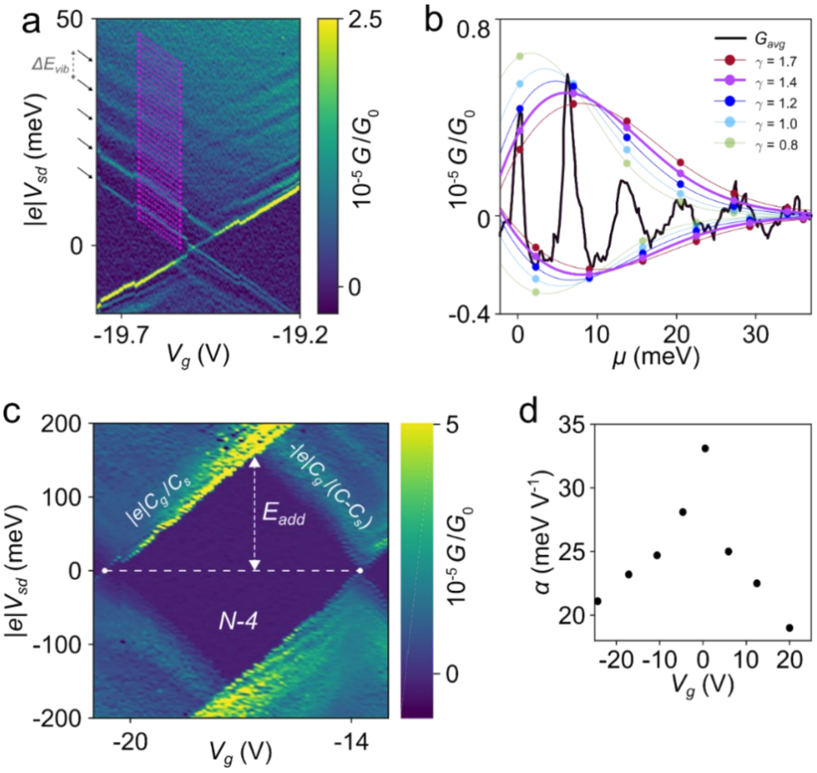

Figure S16: (a) Conductance values along the magenta dashed lines were extracted from the SD and averaged to suppress contributions from conductance ridges that are not parallel to the diamond edge. (b) The averaged conductance values, G_{avg}, from (a) are plotted as a function of $\mu = |e|V_{sd}$ after subtraction of the linear part of G_{avg}. Curves of $G_{n,0}^{max}$ (see Eq.S1) with peak spacing $E_V = 7$ meV are plotted for different values of γ. $\gamma = 1.4$ yields a good fit of our data. (c) Visualisation of the following quantities: E_{add} , $|e|\, C_g/C_s = \alpha_+$ and $-|e|\, C_g/(C - C_s) = \alpha_-$ on a stability diagram showing the Coulomb diamond of the N-4 charge state. (d) Plot showing the dependency of the level arm, α, on V_g for the different Coulomb diamonds in Figure 3d (main text). The value of α is highest around $V_g = 0$ and decreases nonlinearly on either side.

Ab–initio Calculations. Ab-initio density functional theory calculations of MGNR were implemented with Siesta using PBE GGA functional.[5] Sufficient vacuum distance is added along the non-periodic directions to avoid unwanted interactions. Numerical atomic

pseudopotential was used together with an energy cut-off of 400 Ry. Monkhorst Pack k-point-grid was chosen to be 20 × 1 × 1along the three Cartesian directions. The structure is optimized until the maximum force on the atoms is less than 0.01 eV/Å.

For the phonon calculations, a k mesh grid was chosen to be 20 × 1 × 1 and energy cut-off 400 Ry. The dispersion relation is calculated with a supercell size of (3, 1, 1), where 3 represents 3 unit cells along the periodic direction. Atomic displacement of the Gamma phonon mode is visualized with XCrySDen software (Fig.4e in the main text). We need to point out that the side groups in our calculation are simplified. Since we have to compromise with the computational capability of DFT program, we kept only anthracene structure in the side group.

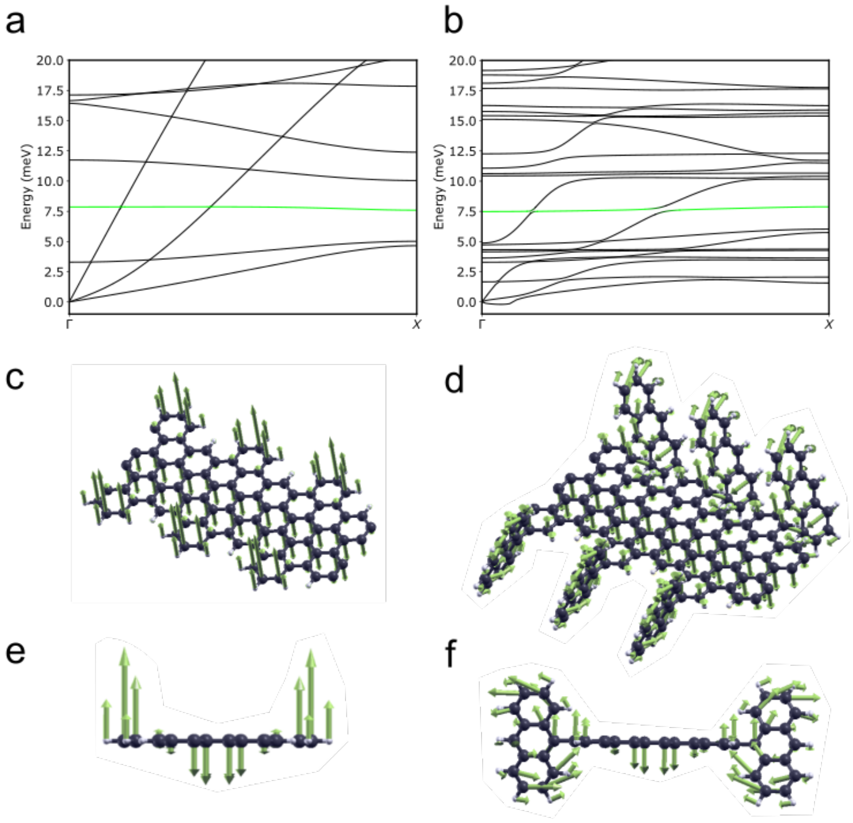

Figure S17: Phonon dispersion relations of MGNR (a) without and (b) with side groups. The phonon modes around 7.5 meV are marked as green in both (a) and (b). (c) and (d) display the displacement vectors of the phonon mode at Γ point for molecule without/with side groups (showing three repetitive unit cells). (e) and (f) are viewed from the longitudinal direction, corresponding to (c) and (d) respectively.

Figure S14 compares the phonon calculation for MGNR without and with side groups. There are many new phonon modes after introducing the side group. Most of the new phonon modes

are localized at the side group, while a small proportion of them are the vibration of MGNR backbone induced by side group. Nevertheless, from the dispersion relation we can find that the phonon mode around 7.5 meV does not change significantly. The energy at Γ point is reduced from 7.86 meV to 7.48 meV. The latter is even closer to the experimental value. This vibrational displacement of this phonon mode is the bending of MGNR backbone. With the side group working like an anchor for the cove edge, the bending amplitude of backbone is suppressed. The displacement of central backbone atoms doesn't change remains similar but the displacement of cove edge atoms is greatly shortened. From this qualitative observation we can estimate that the bending will be mostly confined at the backbone centre if we include the full side group structure into consideration.

Fitting the Conductance Peaks of Vibrational Origin. As explained in the main text, Franck–Condon theory predicts that conductance peak intensities $G_{n,0}^{max}$ associated with a single vibron in equilibrium follow the progression given by Equation S1:

$$G_{n,0}^{max} = \frac{\gamma^n}{n!} e^{-\gamma}, \tag{S1}$$

where n is the vibron quantum number and γ is the electron–vibron coupling. The conductance, averaged along the diamond edge, as explained above, was plotted as a function of chemical potential, $\mu = |e|\Delta V_{sd}$. The linear background of G was subtracted eliminate other contributions than that of the single vibron, such as electronic excited states, density-of-states fluctuations and transport assisted by the environment-associated phonon bath.

$G_{n,0}^{max}$ was fitted using Equation S1 assuming $n = 1, 2, 3, \ldots 6$, γ as fitting parameter and a peak energy spacing of $\mu = 7$ meV. Figure S13b shows these fits for different γ in the range of 0.9 to 1.5. $\gamma = 1.4$ yield the best fit. $\gamma > 1$ implies strong electron–vibron coupling in the MGNR and is manifested by the first peak being lower than the second, i.e. $G_{1,0}^{max} < G_{2,0}^{max}$. This observation implies Franck–Condon suppression of SET though the ground state. We performed such Franck–Condon analyses on different charge states and similar values for γ were found, except for the transition $N - 3 \leftrightarrow N - 2$ where γ was slightly less than unity ($\gamma \approx 0.8$).

Fitting of Conductance Traces. The conductance peaks in the V_{sd} traces were fitted using a Gaussian function. Lorentzian line shapes are characteristic of transport limited by life-time broadening as frequently observed in other single-electron transistors at low temperature[6]. However, our conductance peaks are best fitted with a Gaussian line shape, which is

characteristic for transport limited by temperature-broadening. We note that the small number of measured conductance values for each peak makes it difficult to conclude whether the line shape is of Gaussian or Lorentzian type.

Line Width of Diamond Edge at Different Temperatures. The left edge of the *N*-3 diamond with a small positive bias ($V_g \approx -12.8$ V) was analysed at different temperatures to investigate the effect of temperature on Franck–Condon suppression. The linewidth of the conduction peak at the diamond edge was estimated for the same diamond at three different temperatures, $T = 25$ mK, 0.5 K and 1 K. A number of adjacent V_{sd} traces close to the degeneracy point were averaged and then fitted using the functions in Equations S2 and S3 (Fig.S14). Equation S2 is a Lorentzian that is proportional to lifetime-broadened conductance peaks:[7]

$$G(\mu) \propto \frac{w/2\pi}{(\mu-\mu_0)^2 + (w/2)^2}\ , \tag{S2}$$

where *w* is the FWHM of the conductance peak.

Equation S3 shows the squared hyperbolic cosine function that is proportional to thermally broadened conductance peaks:[6]

$$G(\mu) \propto \frac{1}{4k_BT\ \cosh^2(\mu/2k_BT)}\ . \tag{S3}$$

Figure S15a shows that Equation S2 fits the data well at all temperatures and that *w* increases linearly with *T* from *w*=0.63 meV at 25 mK to *w*=0.98 meV at 1 K. Figure S15b shows that the data also fits well to Equation 3 but with an extracted *T* that is too high, in particular at 25 mK where the electron temperature and the extracted value of *T* differs by two orders of magnitude. Therefore, we conclude that transport is dominated by lifetime broadening of the MGNR energy levels at low temperature where *w* is the coupling energy to the leads. We expect thermal broadening to become dominant at around 3 K.

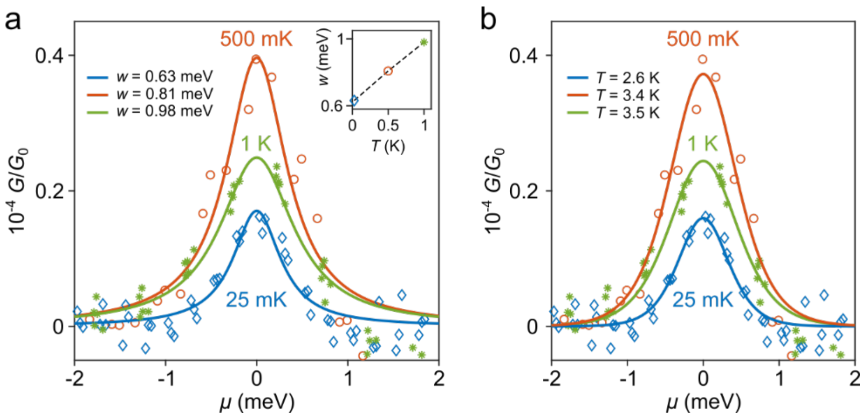

Figure S18: The conductance at a Coulomb diamond edge as a function of $\mu = |e|V_{sd}$ at different temperatures fitted with (a) Eq.S2 and (b) with Eq.S3. (a) Lorentzian line shapes fit the data well for all values of *T* and yield values of *w* ranging from 0.63 to 0.98 meV. The inset shows that *w* increases linearly with *T* up to 1 K. (b) The thermal broadening function fits the data well at all temperatures but the extracted values of *T* are much larger than the electron temperature in the experiment.

V_g-deviation of Conductance Peak Intensities. We observed some V_g dependency on the diamond-edge conductance-peak intensities. Figure S16 shows that G^{max} of the first two peaks of a V_{sd} trace (analogous to the two first peaks in Figure S13b) depend on V_g, V_{sd} or both. This phenomenon is particularly visible at T = 0.5 K where the two peaks are approximately equal at low V_{sd} but shows an increasing difference up to about V_{sd} = 10 mV where it reaches a saturation of around $4 \cdot 10^{-5}\ G/G_0$. There also seem to be some V_g dependence to G^{max} but it is unclear from our results whether this effect influences the difference. Note that the larger the difference (i.e. 2nd peak larger than the 1st peak) the larger the γ. Therefore, it is important to consider these observations when performing the Franck–Condon analysis. Thus, we chose to only analyse V_{sd} traces taken in the V_g range where the difference between the peaks are approximately saturated.

Moreover, these observations shed light on an obvious limitation of this study, i.e. that we have analysed V_{sd} traces with different V_g lying adjacent on the stability diagram. Although we adjusted the traces with respect to chemical potential before averaging, our method fails to eliminate V_{sd} dependent contributions to conductance. Therefore, we suggest that future studies employ a more robust method where for each *T*: (i) multiple V_{sd} traces are taken at the

same V_g before averaging and (ii) V_g should be chosen so that the 1st peak (diamond edge) occurs at the same V_{sd} for all T.

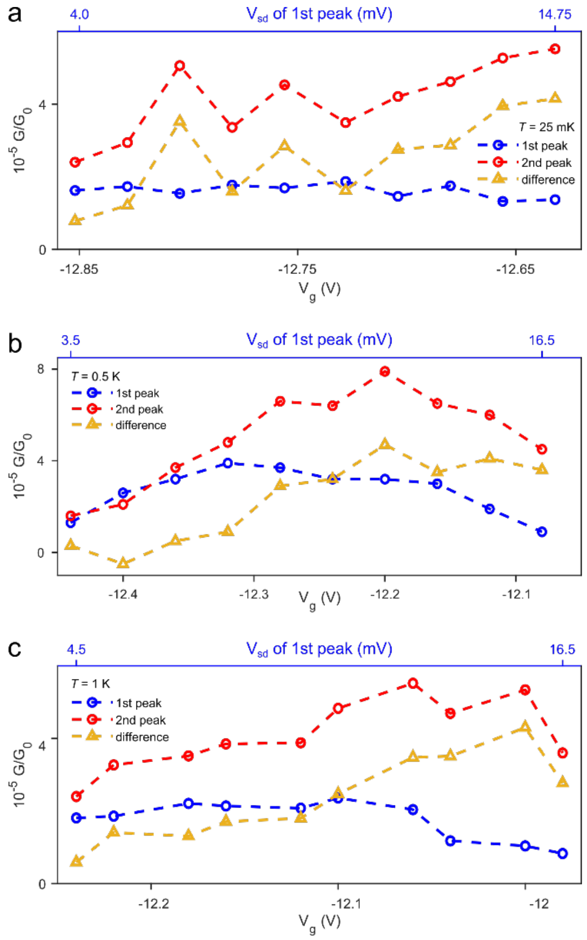

Figure S19: Analysis of conductance peak intensities in V_{sd} traces at different V_g at different temperatures. The figures show G^{max} of the 1st peak (diamond edge) (blue), the 2nd peak (red) and the difference between them (yellow) at temperatures (a) T = 25 mK, (b) T = 0.5 K and (c) T = 1 K.

Estimation of Addition Energies. The addition energy, $E_{add} = E(N) - E(N-1)$, is defined as the energy difference between two MGNR charge states. We estimate an average $E_{add} = 156$ meV from taking the energy from where the edges of the Coulomb diamonds meet and close, averaged over all the measured diamonds (Fig. S13c). Alternatively, the addition energy can be defined as the sum of the charging energy, E_C, and the molecular electronic energy level spacing, ΔE. We observe $E_C \gg \Delta E$ and therefore $E_{add} = E_C + \Delta E \approx E_C$.

Estimation of MGNR Length. We assume that the MGNR is a rectangular quantum dot with width, $w = 1.12$ nm, which length, l, can be estimated as follows:[8]

$$l = \frac{e^2 \ln(4h/w)}{4\pi\epsilon_0\epsilon_r E_{add}}, \tag{S4}$$

where e is the elementary charge, ϵ_0 is the permittivity of free space, $\epsilon_r = 3.9$ is the relative permittivity of the SiO_2 layer[9] of thickness, $h = 300$ nm is the distance between the gate electrode and the MGNR. Using Equation S4 we estimate $l = 33$ nm for **2**, and various lengths (l =28, 90, 40, 17, 108, 60, 50, 33) for the several MGNRs of **1**.

Estimation of Lever Arm. The lever arm, α, provides the relationship between V_g and $|e|V_{sd}$ and can thus be used to convert a change in V_g to the chemical potential, μ. α is associated with the slopes of the Coulomb diamonds which are functions of the capacitances, C_g, C_s and C_d, between the quantum dot (MGNR) and the gate, source and drain electrodes, respectively, and $C = C_g + C_s + C_d$. For negative slopes $\alpha_- = -|e|\, C_g/(C - C_s)$ and for positive slopes $\alpha_+ = |e|\, C_g/C_s$. Our measurements show that the slopes of the diamond edges are approximately symmetric, i.e. $C_s \approx C_d$. Therefore, $\alpha = \alpha_-, \alpha_+$ can be approximated geometrically through $\alpha = E_{add}/\Delta V_g$ where ΔV_g is the width of the Coulomb diamond in units of V_g (i.e. volt). Using this method, we found that α differs for different diamonds in a way that seems to depend on V_g (Fig. S13d). α is largest around small values of V_g and declines symmetrically on either side of $V_g = 0$. This feature indicates that either C_g decreases or C_s increases with increasing $|V_g|$.

Calculation of Stability Diagram. To verify our conclusions, we used a quantum rate-equation model to calculate a stability diagram following the procedure reported elsewhere[5,10]. The model assumes a single vibrational mode with energy $E_V = 7$ meV and a superohmic phonon bath. We assume that the single mode originates from the MGNR and that the phonon bath originates from the environment (dominantly the substrate) to which the MGNR is coupled. The expression for the current through a weakly coupled molecular junction is

$$I = |e| \frac{\gamma_{ox}^{S}\gamma_{red}^{D} - \gamma_{red}^{S}\gamma_{ox}^{D}}{\gamma_{red}^{S} + \gamma_{red}^{D} + \gamma_{ox}^{S} + \gamma_{ox}^{D}}, \tag{S5}$$

where $\gamma_{red/ox}^{S/D}$ denotes the diabatic rates of electron transfers at each electrode (*S*: source; *D*: drain) corresponding to a reduction (red) or an oxidation (ox) of the MGNR. Furthermore, for *N*/*N*+1-type transitions, the rates are given as follows:

$$\gamma_{red}^{S,D} = 2\frac{\Gamma_{S,D}}{\hbar}\int f_{S,D}(\epsilon)k_{red}(\epsilon)d\epsilon\,, \tag{S6}$$

$$\gamma_{ox}^{S,D} = \frac{\Gamma_{S,D}}{\hbar}\int \left(1 - f_{S,D}(\epsilon)\right)k_{ox}(\epsilon)d\epsilon\,, \tag{S7}$$

where $\Gamma_{S,D}$ is the electronic coupling between the MGNR and the source and drain leads, respectively. $f_{S,D}(\epsilon)$ is the Fermi-Dirac distribution in the respective electrode, ϵ is the chemical potential and $k_{red/ox}(\epsilon)$ is the density of states of the MGNR written as:

$$k_{red/ox}(\epsilon) = \frac{1}{\pi}\mathrm{Re}\int_0^{\infty} e^{\sigma i(\epsilon-\mu)t/\hbar}e^{-t/\tau}B(t)dt\,, \tag{S8}$$

where $\sigma = 1$ for reduction and $\sigma = -1$ for oxidation, μ is the molecular energy level, t is time, $\tau = 2\hbar/(\Gamma_S + \Gamma_D)$ is the lifetime of the MGNR electronic state and $B(t)$ is the phononic correlation function:

$$B(t) = e^{D_q(t)} \cdot e^{D_{bath}(t)}, \tag{S9}$$

$$D_q(t) = \left(\frac{g_q}{\omega_q}\right)^2\left[\left(\cos(\omega_q t) - 1\right)\cdot\coth\left(\frac{\omega_q}{2k_BT}\right) - i\sin(\omega_q t)\right], \tag{S10}$$

$$D_{bath}(t) = \int\frac{J(\omega)}{\omega^2}\left[(\cos(\omega t) - 1)\cdot\coth\left(\frac{\omega}{2k_BT}\right) - i\sin(\omega t)\right]dt, \tag{S11}$$

where D_q is the single-mode contribution and D_{bath} is the contribution from the superohmic bath. g_q is the electron-vibration coupling strength, ω is the vibron frequency, k_B is the Boltzmann constant and the spectral density, $J(\omega)$, for the superohmic phonon bath with reorganisation energy, λ, and cut-off frequency, ω_c:

$$J(\omega) = \frac{\lambda}{2}\left(\frac{\omega}{\omega_c}\right)^3 e^{-\omega/\omega_c}. \tag{S12}$$

Using this model we reproduced our experimental data by using the following parameters:

Table S1: Parameters used with Eq. S5-S12 to calculate the stability diagram shown in Figure S17b.

T (mK)	ω_q (meV)	g_q (meV)	ω_c (meV)	λ (meV)	C_S	C_g	Γ_S	Γ_D
25	7	8.5	20	150	0.54	0.025	$1.2\cdot10^{-6}$	$5\cdot10^{-4}$

We assume that the molecular energy level, $\mu = C_S V_{sd} - C_g V_g$, shifts with bias and gate voltages. The calculated conductance $G = dI/dV_{sd}$ was plotted as a function of V_{sd} and V_g and is displayed next to an experimental stability diagram in Figure S17. The calculated and experimental stability diagrams match each other well. Importantly, the calculated diagram shows that the 'single-mode + environment' model reproduces the main features of our experimental data, i.e. conductance lines equally spaced by $E_V \approx 7$ meV, a significant contribution from the phonon bath and conductance asymmetry with bias. The calculated stability diagram contains additional conductance sidebands in the blocked regions that are not present in the experimental diagram.

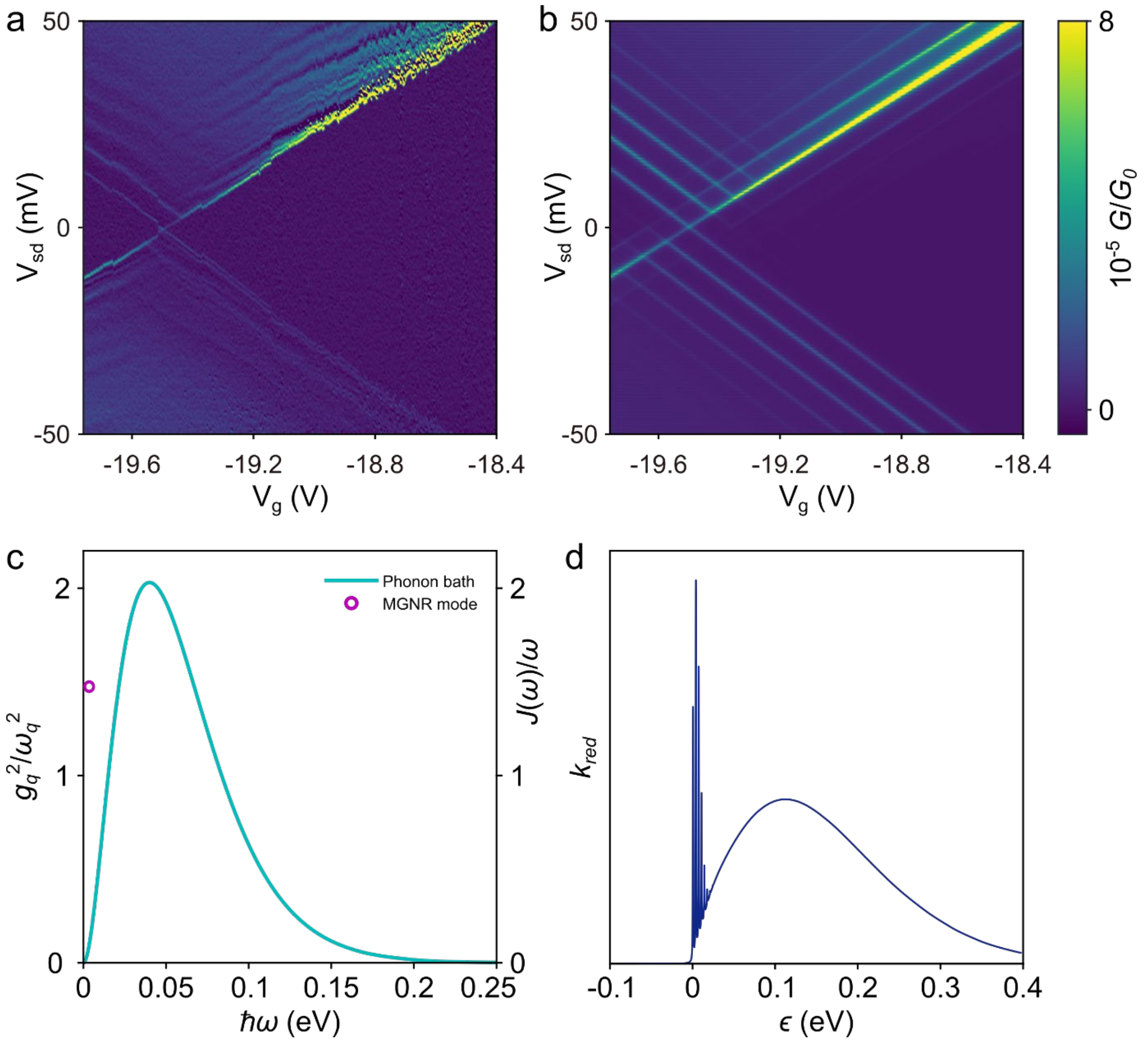

Figure S20: Stability diagrams (conductance map as a function of V_{sd} and V_g plotted using the same conductance colour scale. (a) The same experimental data as in Figure 4 (main text)

plotted in a larger V_g window. (b) Stability diagram calculated using the method discussed above (Eq. S5-S12) with the parameters from Table S1. (c) Calculated spectral densities of the phonon bath (right axis) and the single MGNR mode (left axis). (d) Molecular density of states, k_{red}, as a function of ϵ (from Eq. S8).

Bibliography

[1] Mallik, A.K., Sawada, T., Takafuji, M. and Ihara, H.. Novel approach for the separation of shape-constrained isomers with alternating copolymer-grafted silica in reversed-phase liquid chromatography. *Analytical chemistry*, **82(8)**, 3320-3328, (2010).

[2] Narita, A., Feng, X., Hernandez, Y., Jensen, S.A., Bonn, M., Yang, H., Verzhbitskiy, I.A., Casiraghi, C., Hansen, M.R., Koch, A.H. and Fytas, G. Synthesis of structurally well-defined and liquid-phase-processable graphene nanoribbons. *Nature chemistry*, **6**, 126 (2014).

[3] Lau, C. S., Mol, J. A., Warner, J. H. & Briggs, G. A. D. Nanoscale control of graphene electrodes. *Phys. Chem. Chem. Phys.* **16**, 20398-20401 (2014).

[4] Weig, E.M., Blick, R.H., Brandes, T., Kirschbaum, J., Wegscheider, W., Bichler, M. and Kotthaus, J.P.. Single-electron-phonon interaction in a suspended quantum dot phonon cavity. *Physical review letters*, **92(4)**, 046804 (2004).

[5] Soler, J.M., Artacho, E., Gale, J.D., García, A., Junquera, J., Ordejón, P. and Sánchez-Portal, D.. The SIESTA method for ab initio order-N materials simulation. *Journal of Physics: Condensed Matter*, **14(11)**, 2745 (2002).

[6] Thomas, J.O., Limburg, B., Sowa, J.K., Willick, K., Baugh, J., Briggs, G.A.D., Gauger, E.M., Anderson, H.L. and Mol, J.A.. Understanding resonant charge transport through weakly coupled single-molecule junctions. *Nature communications*, **10(1)**, 1-9 (2019).

[7] Datta, S.. *Quantum transport: atom to transistor*. Cambridge University Press (2005).

[8] Shylau, A.A., Kłos, J.W. and Zozoulenko, I.V.. Capacitance of graphene nanoribbons. *Physical Review B*, **80**, 205402 (2009).

[9] Robertson, J.. High dielectric constant oxides. *The European physical journal applied physics*, **28(3)**, 265-291 (2004).

[10] Sowa, J.K., Mol, J.A., Briggs, G.A.D. and Gauger, E.M.. Beyond Marcus theory and the Landauer-Büttiker approach in molecular junctions: A unified framework. *The Journal of chemical physics*, **149(15)**, 154112 (2018).
